# Dental Diagnosis

**Published:** 1893-12

**Authors:** C. N. Peirce


### DENTAL DIAGNOSIS.[1]

BY DR. C. N. PEIRCE.

In speaking upon dental diagnosis, let us first endeavor to define what it should embrace. The successful study of any subject necessitates its division or arrangement, as far as possible, into branches. In this, one of these should comprise the laws and facts common to individual affections,—that is, whether due to inherent or external influences, and, indeed, everything that can be gathered relating to their causes; and a second should embrace the method of detection and a full recognition of the nature of the affection, its expression, and its influence.

Following in this line or with this purpose in view, you can readily see how diagnosis becomes partly a science and partly an art. A science, because it involves a knowledge of the evolution of the structures and the laws governing their disintegration as well as their integration. An art, because it demands a delicacy of touch, an accuracy of observation, and a quickness of perception; qualities which can only be gained by experience, by practice, and by thorough mental training. Hence I cannot presume to talk intelligently upon this important phase of our daily practice without making first an inquiry regarding the natural forces which have been instrumental in establishing the shapes and density, as well as the development, of the structures with which we have to deal.

The great naturalist Linnæus wrote as early as 1784 that the stony rocks are not primeval, but the daughters of time, and since then geologists, astronomers, zoologists, and scientific men generally have declared that not only this our earth, but all growths and things thereon, together with the planets and stars, have only come

---

[1] Read before the Woman's Dental Association of the United States, October 7, 1893.

into their present liquid and solid condition after immense intervals of time, and hence very gradually. They believe that each and all existing forms are wholly the result of surrounding conditions and forces which were favorable to their development. That slowly and very gradually have the animal as well as the vegetable growths been evolved from pre-existing waters and rocks. That all forms and structures at the present time existing have only been produced when the environment favoring such growths enabled the forces of nature to call them forth.

To impress upon you the influence of environment, I have but to remind you of the facts you have gained from your previous studies in your chemical and physiological laboratories; the combination of the same materials under different conditions and in different proportions producing such varied results, and how differently the same degrees of heat act on various liquids and solids. Our physiologists will tell us that organic structures as well as inorganic are sensitive to varying influences, and give under different circumstances just as great an array of modified and dissimilar results. That every manifestation of force by a muscle or a nerve is the result of a transformation of the structure or of its substance, varying with the effort; that every conception, every mental affection, every sensation, is followed or accompanied by a change of the composition of the substance of the brain. That sensations and movements are simple or complex in proportion as the structures themselves are homogeneous or heterogeneous, and that the morbid or abnormal conditions manifested in these structures vary in the same ratio.

Now, what we first need to appreciate is that the dental structures are in their origin and evolution not in the least different from all other tissues; that they have come into their present pathological as well as physiological condition by a long and slow process and in response to natural forces; that their history, if written out, would be like unto that of all other tissues or organs. When we recognize visible signs of dental decay or premature loss, so patent to our eyes and sense of touch, we want to appreciate the fact that this superficial condition, which is a disturbance of what was but a short time before apparently normal tissue, as recognized by our unassisted senses, is not the complete revelation, it is but the local expression of some near or remote cause, for the discovery of which science has lent and is lending us its aid. The true surgeon or physician is not content with the treatment of superficial manifestations, but with chemical, microscopical, and physiological knowledge, he, if possible, goes beyond the surface, to see if there is not some lurking vice—nutritive or constitutional—underlying the local expression. In like manner must the educated dental specialist look for some departure from normality at some remote time and in remote structures or organs which have induced constitutional predispositions or local affections. As an illustration. A change has taken place. A change of what? The result is observed, the lesion described with definiteness and accuracy. We give its location, the structures involved, and appreciate the probable complications which would beset us in our efforts at arrest and restoration. It may be, or we will assume it is, a simple case of dental decay, with limited disintegration of the mesial or masticating surface, or both combined, of a molar or bicuspid; we go so far as to state that this disintegration is preceded by death of the part, and that the solvent was an acid, the source of this agent being the saliva, and that its virulence was augmented by the presence of certain micro-organisms. What then? Have we answered the query as to the cause of the anomaly? Probably not. The symptoms of the malady we have recognized, as well as some of the sequences, but what do we know of the relations of these sequences to the diseased or to the normal tissues, or of the predispositions or of the cause of the predispositions? Let us remember that the study of any phenomena, whether normal or abnormal, only reaches its final and legitimate conclusion when we have traced it back to its first departure, with a history of the circumstances which were present and could exert a modifying influence.

If structural predispositions are suggested or recognized, did these have their origin in constitutional or local peculiarities? If the former, did they arise from family, society, or racial methods or customs? Certainly the study of any abnormal expression should not be ended until the influence of all these previous or preceding conditions has been, as far as possible, ascertained.

We see what a gauntlet every organ and tissue has to run, subjected to modifications from use or disuse, environment, nutrition, and whim, from or through a line of ancestors not equalled in age by civilization, or, indeed, by the age of man himself. Every change in the actions of life must have had its influence on the growth and development of every organ and structure. While there is complete correspondence or harmony between structure and function modifications would be at their minimum, but when upon organs and tissues a continued strain has been brought, or the reverse of this, a diminution of force, then there must result as a natural consequence either atrophy, disintegration, or integration. Rest or equilibrium in life we know is almost an imaginary factor. The ideally perfect state must be wholly a fancy. Life with an organism whose inherent force is so exactly adjusted to external influences as to remain immobile and impassive under varying states would be a crystallized being, motionless and emotionless.

The only living existence of which we are cognizant is in a perpetual struggle, and its greatest gain is that advantage which a temporary harmonious adjustment gives the inherent over the exterior forces, and with this such opportunity as enables the unit to repair its losses or produce another unit, to take up the struggle and perpetuate itself through an infinite series, and so reap the physiological profit which is to be gained by inheritance or transmission.

Through all these successive complications we do have established types and typical characteristics, abnormal as well as normal, and these are sufficiently permanent and uniform in character to be similarly modified by like influences, inherent as well as exterior. As there is, then, a certain consecutiveness in the order of development, so must there be disease occurring and even recurring in conformity with the fixedness of types and similarity of conditions.

Every abnormal or pathological condition we now see rests on three factors: first, the primary injury representing the incident or external force; second, the susceptibility of the part, which is the representative of the internal or inherent force; and, third, the pathological effect, which is the result. In pathological studies neither of these factors can be ignored.

It is recognized that susceptibility to disease has its origin in defects of development; also that these defects are not peculiar to the individual, but occur in greater or less degree in the same form throughout a large portion of civilized people; this being the case, we must assume that a similarity in development, with a similarity in environment, must produce a like susceptibility and a remarkable sameness in the expression, which we recognize as disease. But we must remember that susceptibility to disease is ever changing or shifting with the stage of development, or with the age of the patient; the weak points differ at different periods of life, and this is especially true with the dental organs.

The dental practitioner who undertakes to relieve disease, or the abnormal conditions for which advice or attention has been solicited, without understanding or at all appreciating the conditions which have induced the abnormality, is groping in the dark; and while success may at times favor the methods adopted in the end, it falls far short in satisfaction of a practice based upon a knowledge of the cause, with an intelligent reason for each step in the operation. In utter disregard, however, of all our studies and desires, we meet with most serious obstacles, from the fact that frequently several lesions coexist; disease is very often a complex state, and when one portion of the economy gets out of order others are apt to follow; hence it follows that a diagnostician, to be reliable, must be an anatomist, so as to pronounce on the seat of the malady; must be a physiologist, so as to appreciate the aberration of functions; and, above all, must be a pathologist, so as to understand the antagonism between diseases, the frequency with which they coexist, their numerous and varied expressions, and the influence of remedial agents upon them.

As an illustration of the influence of force, and also of the possible retrogression or degeneration of structures from its absence or withdrawal, let us examine the inferior maxilla, its attachment, power, and its movements. As you are all aware, it is simply a lever, and one of the third class, where power is between the fulcrum and the weight; the condylar attachment with the glenoid cavity being the fulcrum; the muscles—masseter, buccinator, and temporal—being the power applied to the anterior part of the ramus, or its locality just anterior to the fulcrum, and the jaw anterior being the weight. The power of these muscles as exerted on the movable inferior dental arch against the fixed superior arch is beautifully shown in its varied influences, the differences in the sizes of the opposing surfaces representing the greater or lesser forces exerted upon the molars, bicuspids, and incisors, as they are near or remote from the point of applied power. Assuming that the presence of the enamel, as well as its density, is the result of the resistance offered by the food of the ancestral animal and man, and of the necessary power for the crushing and trituration or comminution of that aliment, then the withdrawal or lessening of that force by the change of diet must in time not only lessen the capacity of the muscles for exerting that maximum power, but would also lessen the capacity of the structures to withstand the greater resistance which had been necessary in the preparation of the tougher and harder nutriment. So here we have an illustration of how the diminution of force can induce a predisposition to atrophy or disintegration, if not the actual result itself.

Let me next give an illustration of the coexistence of diseases which may cloud or embarrass our diagnosis and complicate our treatment. I mention now a case with which I have been confronted on more than one occasion. The patient has an impacted inferior third molar with superficial ulceration of overlying soft tissue, as well as accompanying degenerative processes going on around the roots from the inflammatory condition; concomitant with this local condition severe gastric trouble from some cause is established. A most distressing condition follows. The gingival borders encircling the lower jaw become the seat of severe inflammation and ulceration. This same condition the writer had from local irritation induced by partial superior denture, only it was located upon the superior arch instead of the inferior.

The presence of any irritating local condition, which may be induced by *salivary* or *sanguinary calculus*, dental decay, deep or superficial, mal-occlusion or non-occlusion of teeth, pericementitis, dead or decomposing pulps, loose or unopposed teeth, we all recognize that the presence of any one of these conditions may induce a trifling or trivial irritation, but any one of them coexisting with certain other morbid states or predispositions, constitutional or otherwise, and the disturbance and suffering would be greatly exalted.

Let us appreciate the fact, then, that to conduct a clinical inquiry with precision, satisfaction, and ease we need to follow some well-digested and arranged plan. If we have not this, we may wander about, jumping at very unsafe or unsatisfactory conclusions.

